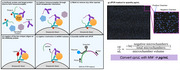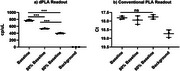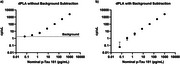# Absolute quantitation of protein enabled by digital proximity ligation assay to enhance quality control of protein reference materials

**DOI:** 10.1002/alz70856_099863

**Published:** 2025-12-24

**Authors:** Jason Wan, Robert Lin, Hien Nguyen, Paul Hung

**Affiliations:** ^1^ Taudia, PALO ALTO, CA, USA

## Abstract

**Background:**

Protein reference materials play a vital role in generating standard curves for numerical output of immunoassays. However, quality control (QC) of protein reference materials to ensure accurate quantitation and lot‐to‐lot consistency remains a significant challenge. Current approaches in the field depend on relative quantitation against certified standards or spectroscopic methods without a true understanding of the actual quantity of material present. Here we present a novel approach termed digital Proximity Ligation Assay (dPLA) combining Proximity Ligation Assay (PLA) with Digital PCR (dPCR) for QC of protein reference materials, leveraging the absolute quantification nature of dPCR for the best‐in‐class quantification precision and accuracy.

**Method:**

This approach follows the conventional PLA method to conjugate unique oligonucleotide sequences to the antibody pair. The antibody pair is then incubated with the target reference material. After the formation of an immunocomplex of two antibodies and the target, the ligated sequence is heat extracted and quantified using dPCR (Figure 1). In addition, dPCR allows the background signal to be directly subtracted to calculate the actual number of intended binding events, potentially improving linearity and sensitivity. To prove both concepts, pTau181 is used due to its wide commercial availability. The pTau181 standard materials were obtained from Thermo Fisher Scientific, quantified by dPLA, and compared to conventional qPCR readout.

**Results:**

In comparison to the conventional PLA, dPLA can differentiate small changes in signal (Figure 2). The same pTau181 standards at baseline, 80% dilution, and 60% dilution were quantified in three different runs. The dPLA showed within 3% CV for each standard whereas the conventional readout cannot differentiate the diluted concentrations. We then performed 5x serial dilution of the baseline standard. With background subtraction, the linearity is improved, with the potential to quantify the least concentrated standard (Figure 3).

**Conclusion:**

Using dPLA, reference materials are quantified in an absolute manner, enhancing reproducibility across different lots and ensuring consistent and reliable measurements. This method can also be used to minimize instrument‐to‐instrument variation caused by standard materials variations, potentially allowing diagnostic platforms to be deployed widely with superior lab‐to‐lab consistency.